# Supplementary material for: MetaRibo-Seq measures translation in microbiomes
Source: Nat Commun. 2020 Jun 29;11:3268. doi: 10.1038/s41467-020-17081-z (PMC7324362; doi:10.1038/s41467-020-17081-z)
Supplement: Supplementary file 10 — Supplementary Data 7 [file 41467_2020_17081_MOESM10_ESM.zip › File2/Confidence_VeryHigh_Taxonomy/241577_out.krona.html]

Javascript must be enabled to view this page.

members
magnitude
magnitudeUnassigned
count
unassigned
taxon
rank

241577\_out

19

superkingdom
2
19

1239
19
phylum

class
186801
18

18
186802
4
order

SRS013965\_contig\_number\_40709SRS048060\_contig\_number\_contig-100\_1432.79939SRS104636\_contig\_number\_contig-100\_23106.114960SRS142599\_contig\_number\_30964

family
31979
9

9
1485
genus

1776383
1
species

SRS043411\_contig\_number\_5714


SRS075984\_contig\_number\_contig-100\_2182.163778SRS142542\_contig\_number\_19329
species
1262770
2

species

SRS019068\_contig\_number\_12923
2293040
1

1506
2

SRS104636\_contig\_number\_9930SRS147139\_contig\_number\_35862
species

1262784
3

SRS013951\_contig\_number\_19152SRS019161\_contig\_number\_28995SRS1041039\_contig\_number\_3601
species

4
216572
family

genus
459786
4


SRS014979\_contig\_number\_contig-100\_9644.158820SRS076804\_contig\_number\_contig-100\_3065.91502SRS144183\_contig\_number\_contig-100\_2522.211374
species
1947021
3

1897011
1

SRS012273\_contig\_number\_contig-100\_1540.32487
species

1
541000
family

1
292632
genus

1
665956

SRS104311\_contig\_number\_42636
species


SRS104400\_contig\_number\_contig-100\_16067.16067
species
1263002
1
